# Clinical Dose-Response of Inflammation Formula Number 1 Granules Versus Traditional Decoction in the Treatment of Patients With Mild to Moderate Atopic Dermatitis: Protocol for a Multicenter Randomized Controlled Trial

**DOI:** 10.2196/96094

**Published:** 2026-07-03

**Authors:** Ding Li, Xin Ma, Chuanbing Xu, Xin Li, Yang Meng, Wencheng Jiang, Guangpu Luo, Yanping Liu, Fanlingzi Shen, Xiangjin Gao, Ruiping Wang, Bin Li

**Affiliations:** 1 Shanghai Skin Disease Hospital, Baode Road, Shanghai, 200433, China, 86 (021) 36803000; 2 Department of Dermatology, Institute of Dermatology, Yueyang Hospital of Integrated Traditional Chinese and Western Medicine, Shanghai, China; 3 Guangzhou University of Chinese Medicine, Guangzhou, China; 4 Department of Dermatology, The Third People's Hospital of Bengbu, Bengbu, China; 5 Guangzhou Dermatology Hospital, Institute of Dermatology, Guangzhou Medical University, Guangzhou, China; 6 Department of Dermatology, Affiliated Hospital of Gansu University of Chinese Medicine, Lanzhou, China; 7 Institute of Dermatology, Shanghai Academy of Traditional Chinese Medicine, Shanghai, China

**Keywords:** atopic dermatitis, Inflammation Formula Number 1, randomized controlled trial, clinical trial protocol, traditional Chinese medicine

## Abstract

**Background:**

Atopic dermatitis (AD) is a common inflammatory skin disease associated with substantial disease burden. Traditional Chinese medicine (TCM) has shown beneficial effects in improving AD symptoms and reducing recurrence. Inflammation Formula Number 1 (IFN-1), a TCM prescription consisting of 10 herbal ingredients, has demonstrated favorable efficacy in clinical practice. However, traditional decoction preparation is time-consuming and inconvenient for long-term standardized use. Granule formulations may improve convenience, stability, and quality control, but their dose-response relationship and equivalence to traditional decoction remain unclear.

**Objective:**

This multicenter, prospective, randomized equivalence trial aims to evaluate the efficacy, safety, and dose-response relationship of IFN-1 granule formulations compared with traditional decoction in patients with mild to moderate AD.

**Methods:**

A total of 300 patients with mild to moderate AD aged 18 to 60 years will be recruited from 5 hospitals between April 2026 and January 2027. Participants will be randomly assigned in a 2:2:1:1 ratio to the traditional decoction group (n=100, 33.3%), standard-dose granule group (n=100, 33.3%), two-thirds dose granule group (n=50, 16.7%), or one-tenth dose granule group (n=50, 16.7%). All groups will receive 4 weeks of treatment. The primary end point is the proportion of patients achieving at least 50% improvement in the Eczema Area and Severity Index (EASI) at week 4. Secondary outcomes include EASI75, EASI90, Investigator Global Assessment score of 0 or 1, body surface area involvement, Dermatology Life Quality Index, pruritus numerical rating scale, Patient-Oriented Eczema Measure, and TCM syndrome scores. Outcomes will be assessed at baseline and weeks 1, 2, and 4. Statistical analyses will be conducted using SAS (version 9.4). Equivalence will be evaluated using a 2-sided 95% CI approach with a predefined equivalence margin of −15% to +15%.

**Results:**

Funding for this study was obtained in 2025. Ethical approval was granted by the Ethics Committee of Shanghai Skin Disease Hospital in December 2025, and the trial was registered with the International Traditional Medicine Clinical Trial Registry (ITMCTR2026000452). Participant recruitment is scheduled to begin in April 2026 and continue through January 2027. No patients were recruited at the time of manuscript submission. Data analysis is expected to commence in March 2027, and the primary study findings are anticipated to be published in 2028.

**Conclusions:**

This trial will determine whether IFN-1 granules achieve efficacy comparable to traditional decoction while improving treatment convenience. The findings may provide evidence for dose optimization and standardized clinical application of TCM granule formulations in AD management.

## Introduction

### Background

Atopic dermatitis (AD) is a common inflammatory skin disorder, characterized by eczematous lesions accompanied by persistent and severe pruritus [1]. Epidemiological studies indicated that more than 100 million adults and 100 million children worldwide are affected by AD [2], and its incidence has increased markedly over the past decade both globally and in China [3,4]. Owing to its severe pruritus, AD significantly impairs patients’ quality of life [5]. Consequently, AD is considered one of the nonfatal skin disorders associated with the highest disease burden [6].

Currently, the treatment of mild to moderate AD is still primarily focused on topical therapy, which constitutes the foundation of treatment for AD [7]. The commonly used topical treatments include corticosteroids and calcineurin inhibitors, which can effectively suppress local inflammatory responses and relieve skin lesions and pruritus [8]. However, the potential adverse effects of these treatments limit their long-term clinical use [9,10]. Meanwhile, there is still a lack of safe, effective oral treatment options for patients with mild to moderate AD. Therefore, the development of a safe and effective oral therapy for AD treatment could fill a gap in the current treatment landscape for mild to moderate AD.

Traditional Chinese medicine (TCM) offers a feasible solution to the current lack of oral therapies for mild to moderate AD, with demonstrated advantages including safety, efficacy, and a low recurrence rate. On the basis of the TCM principle of “cooling the blood and calming yang,” Inflammation Formula Number 1 (IFN-1) was developed and consists of 10 herbs: *Rehmannia glutinosa* (Sheng Di Huang), *Salvia miltiorrhiza* (Dan Shen), *Saposhnikovia divaricata* (Fang Feng), *Isatis indigotica* (Da Qing Ye), *Angelica sinensis* (Dang Gui), *Sophora flavescens* (Ku Shen), *Scutellaria baicalensis* (Huang Qin), *Prunella vulgaris* (Xia Ku Cao), *Dictamnus dasycarpus* (Bai Xian Pi), and *Glycyrrhiza uralensis* (Gan Cao). The clinical efficacy of IFN-1 in treating AD has been well established, with a favorable safety profile. However, the traditional decoction form, while effective, poses challenges for standardized clinical use due to the time-consuming preparation process. Granule formulations offer advantages in convenience, stability, and quality control.

With the increased clinical use of IFN-1 in granule form, a lack of evidence-based guidance regarding dose conversion and therapeutic equivalence between granules and traditional decoctions has become a key barrier to wider application. On the basis of multicenter intervention data, this study will construct a dosage form dose-response model to define the minimum effective dose and the recommended dose range for granule administration, and to explore stratified application strategies across different health care settings. The results are expected to provide a scientific basis for the standardized use of Chinese herbal granules and the modernization of TCM practice.

### Objectives

This study evaluates the clinical outcomes under standard and reduced dosages of IFN-1 to construct a dosage form dose-response equivalence model and to clarify the efficacy and safety profiles of its traditional decoction and granules. Ultimately, this study aims to establish a scientific framework for the dose-response relationship of TCM compound granules and formulate standardized clinical application guidelines, providing critical data support for the standardized use of TCM.

## Methods

### Study Design

**Figure 1.**
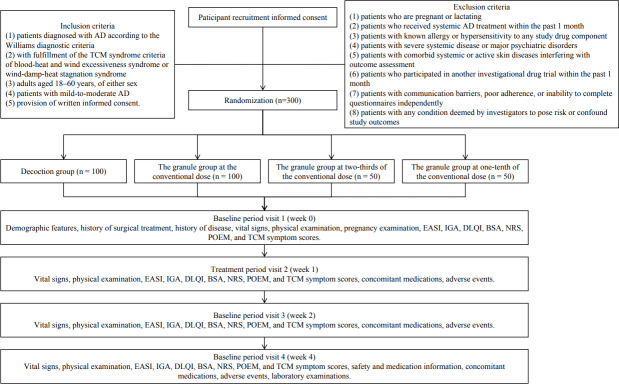
Clinical trial flowchart. AD: atopic dermatitis; BSA: body surface area; DLQI: Dermatology Life Quality Index; EASI: Eczema Area and Severity Index; IGA: Investigator Global Assessment; PNRS: Pruritus Numeric Rating Scale; POEM: Patient-Oriented Eczema Measure; TCM: traditional Chinese medicine.

This study is a multicenter, prospective, randomized clinical trial designed to evaluate the clinical efficacy, safety, and dose-response relationship of IFN-1 administered at a standard dose and reduced doses (granule two-thirds dose and granule one-tenth dose). The study protocol was developed in accordance with the SPIRIT (Standard Protocol Items: Recommendations for Interventional Trials) statement [11]. This study will be conducted according to protocol version 1 (date 2025-10-30). From April 2026 to January 2027, patients with mild to moderate AD will be consecutively recruited from 5 centers, including Shanghai Skin Disease Hospital, Yueyang Hospital of Integrated Traditional Chinese and Western Medicine affiliated with Shanghai University of Traditional Chinese Medicine, Bengbu Third Hospital, Guangzhou Dermatology Hospital, and the Affiliated Hospital of Gansu University of Chinese Medicine. A total of 300 eligible patients with mild to moderate AD will be randomly allocated in a 2:2:1:1 ratio to the traditional decoction group (n=100, 33.3%), standard-dose granule group (n=100, 33.3%), two-thirds dose granule group (n=50, 16.7%), and one-tenth dose granule group (n=50, 16.7%). Patients in all 4 groups will receive treatment for a duration of 4 weeks, during which treatment response and disease progression will be systematically assessed and recorded. The study flow is illustrated in Figure 1.

### Inclusion Criteria

The inclusion criteria for patients with mild to moderate AD are as follows: (1) aged 18 to 60 years, regardless of sex; (2) a clinical diagnosis meeting the Williams diagnostic criteria for AD, with an Eczema Area and Severity Index (EASI) score ≤21 and body surface area (BSA) involvement between 3% and 20%; (3) meeting the TCM diagnostic criteria for wind-damp-heat stagnation syndrome (red, swollen skin with severe burning pruritus, oozing and yellow crusting due to scratching, often accompanied by dry mouth, dark urine, a red tongue with yellow greasy coating, and a slippery-rapid or wiry-rapid pulse; the condition recurs and worsens in hot, humid environments); (4) Investigator Global Assessment (IGA) score ≥3; and (5) provision of written informed consent [12].

### Exclusion Criteria

The exclusion criteria for this study are as follows: (1) pregnant or lactating women; (2) receipt of systemic treatment for AD (including immunosuppressants or biologic agents) within the past 1 month or topical therapy or phototherapy within the past 2 weeks; (3) known allergy or hypersensitivity to any component of the 10 herbs in IFN-1; (4) presence of severe systemic diseases involving the cardiovascular, pulmonary, gastrointestinal, hepatic, or renal systems, or severe psychiatric disorders; (5) presence of systemic diseases or active skin diseases that may interfere with the evaluation of study outcomes, or skin conditions at the target lesion site such as scars or tattoos; (6) participation in another clinical trial within the past 1 month; (7) communication barriers or poor treatment adherence, or inability to independently complete the questionnaires; and (8) any other condition that, in the investigator’s judgment, may pose significant risk to the patient or confound the study results and therefore warrants exclusion.

### Sample Size

This study is designed as an equivalence clinical trial to compare the traditional decoction and the standard-dose granule of IFN-1, with the primary outcome being the proportion of patients with mild to moderate AD achieving EASI50 after 4 weeks of treatment. EASI50 is defined as the percentage of patients achieving ≥50% improvement in EASI score from baseline, reflecting the treatment’s effectiveness in skin lesion improvement. On the basis of previous studies, the EASI50 rate is expected to be 90% in both the traditional decoction group and the granule group at the standard dose. Setting the equivalence margin at Δ=15%, α=.05 (2-sided), and β=.20, the required sample sizes under a 2:1 allocation are 94 and 47, respectively. Considering a 5% dropout rate, the sample sizes are increased to 100 and 50 for the decoction group and the granule standard-dose group, respectively. To explore the dose-response relationship, we include a two-thirds dose granule group (n=50) and a one-tenth dose granule group (n=50). Additionally, 50 patients are added to the standard-dose granule group to strengthen the robustness of the findings. The final study therefore uses a 4-arm parallel design with an allocation ratio of 2:2:1:1 into traditional decoction (n=100), standard-dose granule (n=100), two-thirds dose granule (n=50), and one-tenth dose granule (n=50).

### Randomization

This study is planned to use a stratified block randomization method with “study hospital” as the stratification factor. A total of 300 patients with mild to moderate AD will be randomly assigned in a 2:2:1:1 ratio to the traditional decoction group (n=100, 33.3%), standard-dose granule group (n=100, 33.3%), two-thirds dose granule group (n=50, 16.7%), and one-tenth dose granule group (n=50, 16.7%). The randomization scheme and implementation will be monitored by dedicated statisticians at the Clinical Research Center of Shanghai Skin Disease Hospital. Randomization codes will be generated by the same center and concealed in opaque, sealed envelopes. After recruitment screening, the envelope will be opened by study personnel, and the patient will receive the treatment corresponding to the assigned random number.

### Blinding

This study is designed as an open-label trial, in which both the participants and the investigators will be aware of the treatment assignments. However, the outcome assessors and the statisticians will be blinded throughout the trial period to prevent potential information bias.

### Interventions

In this study, patients in the traditional decoction group, the standard-dose granule group, the two-thirds dose granule group, and the one-tenth dose granule group will receive treatment twice daily, 30 minutes after breakfast and dinner, at a dose of 200 mL for 4 consecutive weeks. All patients will receive baseline therapy with desonide cream combined with urea ointment, applied twice daily for 14 days. After 14 days, desonide cream will be discontinued, and urea ointment will be continued alone for an additional 14 days. The detailed composition of IFN-1 is presented in Table 1, and the dosage of each herbal component in different formulations is shown in Table 2. The IFN-1 granules used in this study were manufactured by Guangdong Efang Pharmaceutical Co, Ltd in compliance with Good Manufacturing Practice. Quality control procedures included authentication of raw herbal materials, testing for heavy metals, pesticide residues, microbial contamination, and batch-to-batch consistency, following the standards of the Chinese Pharmacopoeia (2020 edition).

**Table 1.** Detailed drug composition of Inflammation Formula Number 1 (IFN-1).

Chinese name	Latin name	Pharmacologic activity
Sheng Di Huang	*Rehmannia glutinosa*	Immunomodulation and anti-inflammatory
Dan Shen	*Salvia miltiorrhiza*	Microcirculation improvement and antioxidant
Fang Feng	*Saposhnikovia divaricata*	Antiallergic and analgesic
Da Qing Ye	*Isatis indigotica*	Anti-inflammatory and antiviral
Dang Gui	*Angelica sinensis*	Immunomodulatory and antithrombotic
Ku Shen	*Sophora flavescens*	Antipruritic and anti-inflammatory
Huang Qin	*Scutellaria baicalensis*	Antiallergic, antibacterial, and antifungal
Xia Ku Cao	*Prunella vulgaris*	Antioxidant and immunosuppressive
Bai Xian Pi	*Dictamnus dasycarpus*	Anti-inflammatory and antifungal
Gan Cao	*Glycyrrhiza uralensis*	Steroid-like activity and anti-inflammatory

**Table 2.** Composition and dosage of Inflammation Formula Number 1 in different formulations.

Chinese name	Traditional decoction (g)	Standard-dose granule (g)	Two-thirds dose granule (g)	One-tenth dose granule (g)
Sheng Di Huang	15	15	10	1.5
Dan Shen	9	9	6	0.9
Fang Feng	6	6	4	0.6
Da Qing Ye	9	9	6	0.9
Dang Gui	9	9	6	0.9
Ku Shen	9	9	6	0.9
Huang Qin	9	9	6	0.9
Xia Ku Cao	9	9	6	0.9
Bai Xian Pi	9	9	6	0.9
Gan Cao	6	6	4	0.6

### Follow-Up

All patients with mild to moderate AD will undergo physical examinations, skin lesion assessments, and laboratory tests at baseline (day 0) and at week 4 after enrollment. Treatment efficacy will be evaluated during follow-up visits at weeks 1, 2, and 4. Detailed information on data collection, physical examinations, and skin lesion assessments is shown in Table 3.

**Table 3.** Study schedule of Inflammation Formula Number 1 clinical trial (4 weeks).

Indicator	Baseline visit 1 (week 0)	Treatment visit 2 (week 1)	Treatment visit 3 (week 2)	Treatment visit 4 (week 4)
Informed consent	✓			
Demographic feature	✓			
History of surgical treatment	✓			
History of disease	✓			
Vital signs	✓	✓	✓	✓
Physical examination	✓	✓	✓	✓
Pregnancy test	✓			
Inclusion and exclusion criteria check	✓			
Randomization	✓			
EASI^a^	✓	✓	✓	✓
IGA^b^	✓	✓	✓	✓
DLQI^c^	✓	✓	✓	✓
BSA^d^	✓	✓	✓	✓
PNRS^e^	✓	✓	✓	✓
POEM^f^	✓	✓	✓	✓
TCM^g^ symptom scores	✓	✓	✓	✓
Laboratory examinations	✓			✓
Safety and medication information	✓			✓

a EASI: Eczema Area and Severity Index.

b IGA: Investigator Global Assessment.

c DLQI: Dermatology Life Quality Index.

d BSA: body surface area.

e PNRS: Pruritus Numeric Rating Scale.

f POEM: Patient-Oriented Eczema Measure.

g TCM: traditional Chinese medicine.

### Outcomes

#### Primary Outcome

The primary outcome indicator of this study is the proportion of patients achieving EASI50 after 4 weeks of treatment.

The EASI is used to assess the extent and severity of AD. Four body regions are evaluated separately: head and neck, upper extremities, trunk, and lower extremities. For each region, the percentage of affected area is scored on a 7-point scale (0‐6): 0 (0%), 1 (<10%), 2 (10%‐19%), 3 (20%‐49%), 4 (50%‐69%), 5 (70%‐89%), and 6 (90%‐100%). In each region, 4 clinical signs—erythema, edema or papulation, excoriation, and lichenification—are each graded on a 4-point severity scale (0‐3: none, mild, moderate, or severe). The sum of the 4 signs is multiplied by the area score and a region-specific weighting factor reflecting body surface distribution: head and neck (0.1), upper limbs (0.2), trunk (0.3), and lower limbs (0.4). The total EASI score is obtained by summing all 4 regional scores, ranging from 0 to 72, with higher scores indicating more severe disease. On the basis of the EASI score, the skin lesion condition of patients with AD is categorized as mild (0‐7 points), moderate (7‐21 points), or severe (>21 points). EASI is a static assessment completed independently at each visit. Consistent scoring by the same investigator and proper training ensure accuracy and reliability.

#### Secondary Outcomes

Secondary outcome measures include multiple efficacy and patient-reported outcomes assessed at weeks 0, 1, 2, and 4, specifically the proportions of patients achieving EASI75 (≥75% improvement in EASI score from baseline) and EASI90 (≥90% improvement in EASI score from baseline), the IGA, the Dermatology Life Quality Index (DLQI), BSA, the Pruritus Numeric Rating Scale (PNRS), the Patient-Oriented Eczema Measure (POEM), and changes in TCM syndrome scores from baseline. The complete study protocol, including detailed information on the secondary outcome measures and assessment time points, is presented in Table 3.

##### Investigator Global Assessment

The IGA score is a simplified assessment based on the investigator’s subjective judgment that allows for a rapid evaluation of overall disease severity [13]. At each study visit, investigators assess the patient’s global disease manifestations using a 6-point scale ranging from 0 to 5. The scoring criteria are defined as follows: 0 (clear, no inflammatory signs), 1 (almost clear, minimal residual erythema or papulation), 2 (mild erythema with slight papulation or induration), 3 (moderate erythema with definite papulation or induration), 4 (severe erythema with marked inflammatory lesions), and 5 (very severe, with extensive or confluent erythema and prominent exudation or crusting). One of the outcome measures is the proportion of patients who achieve an IGA score of 0 or 1 with an improvement of at least 2 points from baseline. Longitudinal IGA assessments are performed at baseline (week 0) and at weeks 1, 2, and 4 after treatment initiation.

##### Dermatology Life Quality Index

The DLQI is used to systematically assess patients’ quality of life over the previous week, covering multiple domains including physical symptoms, psychological well-being, family relationships, interpersonal communication, occupational limitations, social activities, and treatment-related impact [14]. The DLQI uses a 4-point severity scoring system for each of the 10 items, resulting in a total score ranging from 0 to 30, with higher scores indicating poorer quality of life. In this study, DLQI will be assessed at baseline (week 0) and at weeks 1, 2, and 4 after treatment initiation.

##### Body Surface Area

BSA is a commonly used clinical indicator for assessing skin disease severity [15]. In this assessment, the area of a patient’s single palm, including the volar surface of the fingers, is defined as 1% of the total BSA. The extent of skin lesions is estimated by calculating how many “palm areas” are involved across the whole body, and changes in the percentage of affected BSA are evaluated relative to baseline. In this study, BSA will be assessed at baseline (week 0) and at weeks 1, 2, and 4.

##### Pruritus Numerical Rating Scale

The PNRS is a single-item patient-reported scale used to assess the severity of pruritus in patients with AD [16]. It uses a 0 to 10 visual analog scale, where 0 indicates no itch and 10 indicates the most severe itch, and patients mark the scale themselves at each visit. In this study, PNRS scores will be assessed at baseline (week 0) and at weeks 1, 2, and 4.

##### Patient-Oriented Eczema Measure

POEM is a repeatable patient-reported outcome measure used to assess the severity of AD [17]. It evaluates the frequency of 7 symptoms experienced over the previous week, including pruritus, sleep disturbance, bleeding, oozing, skin cracking, scaling, and dryness or roughness. Each item is scored from 0 to 4 according to symptom frequency, yielding a total score ranging from 0 to 28, with higher scores indicating more severe disease. In this study, POEM scores will be assessed at baseline (week 0) and at weeks 1, 2, and 4.

##### TCM Syndrome Scores

The TCM syndrome score is assessed using a graded quantitative method based on the local and systemic symptoms of wind-damp-heat stagnation syndrome. The assessment of local symptoms primarily includes the severity of pruritus and the color of skin lesions, each scored on a 4-point scale from 0 to 3, corresponding to absent, mild, moderate, and severe, respectively. For skin lesion sites (head and neck, trunk, upper limbs, lower limbs, and special areas) and skin lesion types (erythema; papules or maculopapules; vesicles; exudation; scales; crusts; hypertrophy, roughness, or lichenification; and hyperpigmentation), each item is scored on a 2-point scale (0‐1), indicating absence or presence. The assessment of systemic symptoms includes acute onset (0=absent, 1=present), floating pulse (0=absent, 1=present), tongue body (0=normal, 1=fresh red, 2=crimson), and tongue coating (0=thin white, 1=thick greasy, 2=yellow greasy). All TCM syndrome assessments will be independently performed by trained dermatologists with a TCM background. A total syndrome score will be calculated, and changes in the score before and after treatment will be compared to evaluate therapeutic efficacy. In this study, TCM syndrome scores will be assessed at baseline (week 0) and at weeks 1, 2, and 4. Details of TCM syndrome scores are shown in Multimedia Appendix 1.

### Safety Assessment

Before enrollment, all patients diagnosed with mild to moderate AD will undergo a standardized informed consent process, during which comprehensive information regarding the study objectives, potential benefits, and possible risks will be fully disclosed. An adverse event is defined as any unfavorable medical occurrence after treatment, including symptoms, signs, diagnosable diseases, or abnormalities in laboratory test results, regardless of whether a causal relationship with the study intervention is established. Participants will report all adverse events occurring during the 4-week treatment period. All reported events will be documented in case report forms (CRFs), together with precise timing and detailed clinical descriptions.

In accordance with clinical practice guidelines, any patients who experience a serious adverse event will be immediately withdrawn from the study and provided with free medical management as specified in the study protocol. These safety measures are designed to ensure rigorous data collection and safeguard participant well-being throughout the trial. Given the minimal risk associated with this study, an independent data monitoring committee will not be established.

### Withdrawal and Dropout

In accordance with the Declaration of Helsinki, participants may withdraw from the study at any time for any reason. All personal information will be kept strictly confidential, and reasons for withdrawal will be recorded in the CRFs.

### Data Collection and Management

This study will adopt a standardized data collection and management procedure. All study data will be collected using CRFs completed by dermatologists who have received unified training. The CRFs consist of six sections: (1) demographic characteristics, including age, sex, date of birth, marital status, and so forth; (2) medical history, including disease history, prior surgical treatments, allergy history, and family history of AD; (3) vital signs and physical examination, including height, weight, and BMI; (4) disease severity and treatment efficacy assessments, conducted at baseline (week 0), week 1, week 2, and week 4, including the EASI, IGA, DLQI, BSA, PNRS, POEM, and TCM syndrome scores; (5) laboratory examinations, including pregnancy testing, routine blood tests, routine urinalysis, and assessments of liver and renal function; and (6) safety and medication information, including prior medication use, concomitant medications, and adverse events.

In this study, a database will be established using EpiData (version 3.1; EpiData Association), with input validation rules defined for each variable, including data type, value ranges, and logical checks. Data entry will be performed independently using a double-entry method, followed by consistency checks between the 2 datasets. Any discrepancies will be resolved by referring to the original CRFs until complete agreement is achieved. The finalized database will be converted into SAS datasets, and data validation and logical checks will be conducted using SAS (version 9.4; SAS Institute) [18].

### Statistical Analysis

Statistical analyses will be performed using SAS (version 9.4). The primary analysis will follow the per-protocol principle, with supportive analyses conducted under the intention-to-treat framework including all randomized patients. Missing data will be handled under the missing-at-random assumption using sequential regression multiple imputation. This study is designed as an equivalence trial, with the primary end point being the proportion of patients achieving EASI50 at week 4. The prespecified equivalence margin (Δ) was set at 15% based on clinical considerations. Equivalence will be assessed using the 2-sided 95% CI approach: the difference in EASI50 response rates between groups will be estimated, and the corresponding 95% CI will be constructed using the chi-square test. Equivalence will be concluded if and only if the entire CI lies within the predefined margin of −15% to +15%. For intergroup comparisons among the 4 treatment groups, an overall test will first be performed. Subsequently, pairwise comparisons will be conducted where appropriate. Between-group comparisons will be performed using the Student *t* test (2-tailed) or Wilcoxon rank-sum test, as appropriate. For multiple pairwise comparisons, Bonferroni correction will be applied to control for type I error. Linear mixed-effects models will be applied for continuous outcomes, and generalized linear mixed models will be used for binary outcomes, with subject-specific random effects to account for within-subject correlation and to appropriately handle missing data. Categorical variables will be expressed as frequencies and percentages and compared using the chi-square test or Fisher exact test. In addition, a dose-response model will be constructed to characterize the dose-response relationship of granules and to explore the formulation conversion between granules and traditional decoction. Except for the equivalence testing described above, a 2-sided *P*<.05 will be considered statistically significant.

### Ethical Considerations

This clinical trial protocol (version 1.0, December 2025) has been approved by the Ethics Committee of Shanghai Skin Disease Hospital (approval 2025‐47) and registered with the International Traditional Medicine Clinical Trial Registry (ITMCTR2026000452). The study will be conducted in strict accordance with the ethical principles outlined in the Declaration of Helsinki and its subsequent amendments. All participants will provide written informed consent prior to enrollment; for participants unable to provide consent themselves, their legal guardians will provide written consent on their behalf.

All signed informed consent forms and collected CRFs will be securely stored in locked facilities and will be accessible only to the study team. All study data will be kept at Shanghai Skin Disease Hospital for 10 years after study completion and will then be destroyed in accordance with applicable regulations.

## Results

Ethics approval and trial registration for this study were obtained in December 2025. Participant recruitment is scheduled to begin in April 2026 and continue through January 2027. Data processing and statistical analyses are scheduled to begin in March 2027. The primary results are expected to be submitted for publication in a peer-reviewed scientific journal in January 2028.

## Discussion

### Anticipated Findings

This study pioneers a dermatology-focused framework to define the formulation dose-response relationship between granules and traditional decoctions of TCM. Focusing on IFN-1, the study aims to evaluate the clinical equivalence and dose-response relationship between its granule formulation and traditional decoction in the treatment of mild to moderate AD. Key issues will be addressed, including formulation conversion, dose-gradient optimization, and safety boundaries, with the goal of providing valuable evidence-based medical data.

Oral TCM has demonstrated notable advantages in the management of AD, including proven clinical efficacy, favorable safety profiles, and suitability for long-term administration [19]. Nevertheless, traditional decoctions of TCM are limited by time-consuming preparation and inconvenience, which hinder their clinical use. Granules, as a modernized form of traditional decoctions, effectively address these limitations and offer advantages such as convenient administration, stable quality, accurate dosing, and a high level of standardization, leading to increasing clinical adoption. However, the dose-response relationship and equivalence between traditional decoctions and granules remain unclear, representing a major barrier to the standardized use and optimization of herbal granules. Clarifying this relationship is essential for maximizing therapeutic efficacy, reducing patient burden, and further promoting the modernization and standardization of TCM.

IFN-1 is a proprietary TCM based on extensive clinical experience. It contains multiple herbal components with anti-inflammatory and antiallergic properties. *Rehmannia glutinosa* has been shown in AD animal models to significantly reduce skin inflammation, decrease lesions and inflammatory cell infiltration, and lower serum immunoglobulin E and histamine levels [20]; *Saposhnikovia* divaricata can modulate immune responses and alleviate skin inflammation [21]; *Sophora flavescens*, through its active compound formononetin, can suppress inflammatory responses and neutrophil activation, thereby reducing pruritus and skin lesions [22]; *Scutellaria baicalensis* exhibits anti-inflammatory and antioxidant effects, improving skin inflammation [23]; *Glycyrrhiza uralensis* contains active compounds such as *glycyrrhizin*, which regulate immune function, enhance the efficacy of the formula, and mitigate irritant effects and adverse effects [24]. On the basis of these pharmacological actions, IFN-1 has been widely used for AD treatment, with substantial real-world evidence supporting its efficacy and safety.

This study has several limitations. First, only patients with AD meeting the TCM criteria for wind-damp-heat stagnation syndrome are included, which may limit the generalizability of the findings to patients with other TCM syndromes or different types of AD. Second, although different dosage granules are included in this study to explore the dose-response relationship, the dosage stratification is primarily based on previous clinical experience and does not fully cover all potential dose ranges. The optimal dose still needs to be further refined in studies with larger sample sizes and more precise dosage designs.

### Conclusions

This study is expected to clarify the dose-response relationship and identify the optimal therapeutic dose that maximizes efficacy while minimizing the treatment burden for AD. The findings are expected to provide important evidence for a safe and effective oral treatment option and will be disseminated through peer-reviewed dermatology and international scientific journals.

## Supplementary material

10.2196/96094 Multimedia Appendix 1 The details of the traditional Chinese medicine (TCM) symptom scale.

10.2196/96094 Checklist 1 SPIRIT checklist.

## Abbreviations

AD: atopic dermatitis
BSA: body surface area
CRF: case report form
DLQI: Dermatology Life Quality Index
EASI: Eczema Area and Severity Index
IFN-1: Inflammation Formula Number 1
IGA: Investigator Global Assessment
PNRS: Pruritus Numerical Rating Scale
POEM: Patient-Oriented Eczema Measure
SPIRIT: Standard Protocol Items: Recommendations for Interventional Trials
TCM: traditional Chinese medicine
