# Supplementary material for: Clinical Dose-Response of Inflammation Formula Number 1 Granules Versus Traditional Decoction in the Treatment of Patients With Mild to Moderate Atopic Dermatitis: Protocol for a Multicenter Randomized Controlled Trial
Source: JMIR Res Protoc. 2026 Jul 3;15:e96094. doi: 10.2196/96094 (PMC13330736; doi:10.2196/96094)
Supplement: Multimedia Appendix 1 [file resprot-v15-e96094-s001.docx]

**Multimedia Appendix 1.** The details of the traditional Chinese medicine (TCM) symptom scale.

| **TCM^a^ Symptom Scale** |
| --- |
| **Local Symptoms (Lesion Characteristics and Pruritus Severity)** |
| 1. **Lesion Location:** □head & neck □trunk □upper limbs □lower limbs □special sites |
| 1. **Lesion Type** |
| Erythema： □No □Yes |
| Papuless： □No □Yes |
| Vesicles： □No □Yes |
| Exudation： □No □Yes |
| Scaling： □No □Yes |
| Crusts： □No □Yes |
| Lichenification： □No □Yes |
| Hyperpigmentation： □No □Yes |
| 1. **Lesion Color：**  □Normal □Brown □Light Red □Bright Red |
| 1. **Pruritus Severity：**□None □Mild □Persistent but tolerable □Severe and unbearable |
| **Systemic Symptoms** |
| **Inquiry** |
| Acute Onset： □Yes □No |
| **Tongue Body** |
| 1. Tongue Body： □Normal □Bright red □Deep red |
| 1. Tongue Coating： □Thin white □Thick greasy □Yellow greasy |
| **Pulse Palpation** |
| 1. Floating Pulse： □Yes □No |
